# Spatial and quantitative datasets of the pancreatic β-cell mass distribution in lean and obese mice

**DOI:** 10.1038/sdata.2017.31

**Published:** 2017-03-14

**Authors:** Saba Parween, Maria Eriksson, Christoffer Nord, Elena Kostromina, Ulf Ahlgren

**Affiliations:** 1 Umeå Centre for Molecular Medicine, Umeå University, Umeå S-90187, Sweden

**Keywords:** Cell biology, Islets of Langerhans, Optical imaging

## Abstract

A detailed understanding of pancreatic β-cell mass distribution is a key element to fully appreciate the pathophysiology of models of diabetes and metabolic stress. Commonly, such assessments have been performed by stereological approaches that rely on the extrapolation of two-dimensional data and provide very limited topological information. We present *ex vivo* optical tomographic data sets of the full β-cell mass distribution in cohorts of obese *ob/ob* mice and their lean controls, together with information about individual islet β-cell volumes, their three-dimensional coordinates and shape throughout the volume of the pancreas between 4 and 52 weeks of age. These data sets offer the currently most comprehensive public record of the β-cell mass distribution in the mouse. As such, they may serve as a quantitative and topological reference for the planning of a variety of *in vivo* or *ex vivo* experiments including computational modelling and statistical analyses. By shedding light on intra- and inter-lobular variations in β-cell mass distribution, they further provide a powerful tool for the planning of stereological sampling assessments.

## Background & Summary

**Figure 1:**
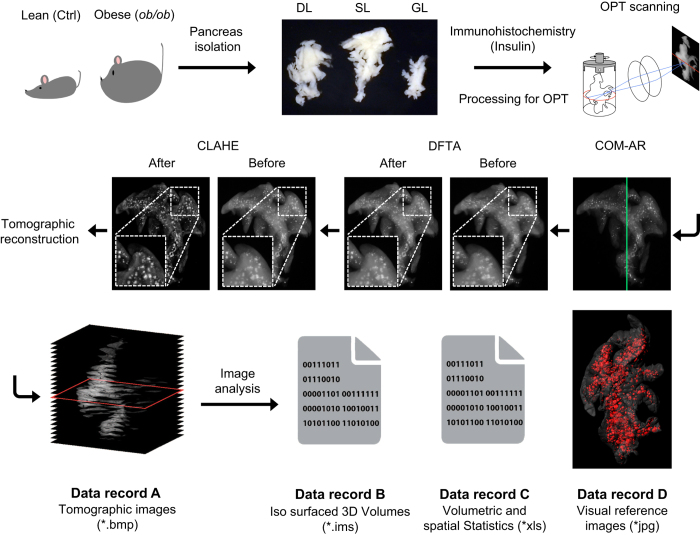
Schematic workflow of the assay design. Pancreata were isolated and divided into the three primary lobular compartments prior to whole mount immunohistochemistry, agarose embedding and tissue clearing. The processed samples were aligned (COM-AR) in the OPT prior to scanning. Two in-house developed image processing scripts were applied to the collected projection views, DFTA (uniforming alignment values) and CLAHE (equalising the contrast of the insulin labelled islets) before they were reconstructed into tomographic images (Data record A, Data Citation 1). The tomographic images were imported into the Imaris software where they were iso-sufaced (Data record B, Data Citation 1). Volumetric and spatial statistics was extracted in Imaris (Data record C, Data Citation 1) and images for visual reference exported (Data record D, Data Citation 1).

**Figure 2:**
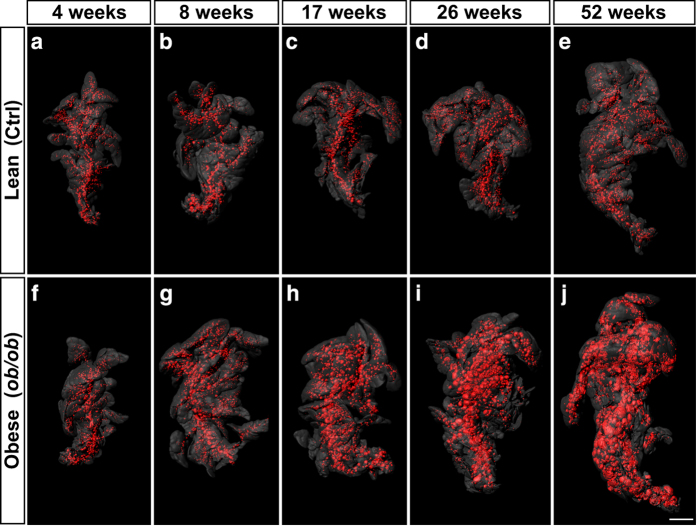
Examples of representative iso-surface rendered OPT images. (**a**–**j**) Islet β-cell distribution in *ob/ob* (**a**–**e**) and lean control (**f**–**j**) pancreata (splenic lobe) between 4 and 52 weeks of age. The islet β-cell volumes are reconstructed based on the signal from insulin specific antibody staining (red) and the pancreas outline (gray) is based on the signal from tissue autofluorescense. Scale bar in (**j**) corresponds to 2 mm in (**a**–**j**).

The pancreas controls multiple homeostatic functions through the activities of its constituent exocrine acinar and endocrine islet cells. The endocrine component, which main function is to control blood glucose homeostasis, comprise a fraction of the pancreatic mass and is organized into the islets of Langerhans. The islets contain five hormone-producing cell-types of which the insulin producing β-cells is the most prominent type (~80% in humans and mice). Given their role in glucose metabolism, the pancreatic β-cells play a key role in the aetiology of diabetes. However, even in a small animal like the mouse, the predominant model system for studies of diabetes and metabolic stress, the ability to obtain a detailed spatial and quantitative view of the pancreatic β-cell mass distribution has been limited. Whereas stereological sampling techniques rely on the interpolation of 2D data for quantitative assessments and provide limited topological information, current non-invasive techniques provide only diminutive topological information and are associated with a number of technological hurdles for quantitative analyses^1,2^. Subsequently, comprehensive records of the natural or pathologic distribution of the pancreatic β-cell mass (here β-cell volume (BCV) will be used since it better adheres to the principle of detection), taking the entire pancreatic volume into account, are essentially lacking. By optical projection tomography (OPT)^3^ imaging protocols developed in our laboratory^4–7^ it is possible to obtain detailed (islet level) information of this kind, throughout the volume of the pancreas in large cohorts of animals^7–10^. Within the academia and the pharmaceutical industry alike, the *ob/ob* mouse model is widely used for studies on initial aspects of metabolic disturbances leading to type 2 diabetes, including insulin resistance and obesity^11^. Further, it is well established that the model displays a dramatic increase in β-cell mass to compensate for increased insulin demand. To address the current lack of combined topological and quantitative information of the BCV distribution in obese (*ob/ob*) mice and their healthy lean controls, we recently assessed the full BCV distribution at 4, 8, 17, 26 and 52 weeks of age by OPT imaging analyses^12^ (for schematic overview of the assay design see Fig. 1). Here we present the data sets on which this study was based, including:

- OPT generated tomographic image stacks (*.bmp) throughout the volume of the splenic, duodenal and gastric lobes of *ob/ob* and control pancreas for the insulin and the anatomy channel.
- Imaris (*.ims) files with iso-surfaced volumes (i.e., segmented islet volumes), here defined as a continuous body of insulin positive cells (i.e., the volume of the insulin producing cells within each islet) based on the signal from insulin specific antibodies, and pancreatic lobular volumes based on the signal from endogenous tissue autofluorescence.
- Excel sheets (*.xls) providing information about the individual islet β-cell volumes, their corresponding 3D coordinates and related aspect ratio information, together with information on pancreatic lobular volumes.
- Representative iso-surfaced reconstruction (i.e., segmented islet volumes) images of each sample for visual reference (*.jpg) (For example see Fig. 2).

The tomographic image stacks (Tomographic images.zip_Data record A, Data Citation 1) may be fed into a number of image analyses software for 3D rendering of 2D image stacks (see Usage Notes below), allowing a range of spatial and quantitative features of the BCV distribution to be analysed. This include assessments of spatial interrelationships (such as intra- and interlobular BCV distribution patterns), the spatial and temporal distribution of (arbitrarily selected) islet size categories, islet and BCV densities or other aspects of BCV distribution or growth dynamics. Using the Imaris (*.ims) files of segmented volumes (Isosurfaced Volumes.zip_Data record B, Data Citation 1), the above types of assessments could be directly performed in the Imaris software without the need for prior data segmentation. Excel sheets (Volumetric and spatial statistics.zip_Data record C, Data Citation 1) comprise numerical data logs displaying information about individual pancreatic lobular and islet β-cell volumes together with their corresponding 3D coordinates and related object parameters. These logs may be used for statistical analyses of BCV distribution patterns, also without the access to advanced 3D imaging/analysis software. Finally, representative 3D iso-surfaced visual reference images corresponding to all specimens included in the study are made available (Visual reference image.zip_Data record D, Data Citation 1). Jointly, these data records may further provide reference material for the planning and execution of stereological sampling assessments or established or experimental non-invasive imaging approaches.

## Methods

### Animals and organ isolation

**Figure 3:**
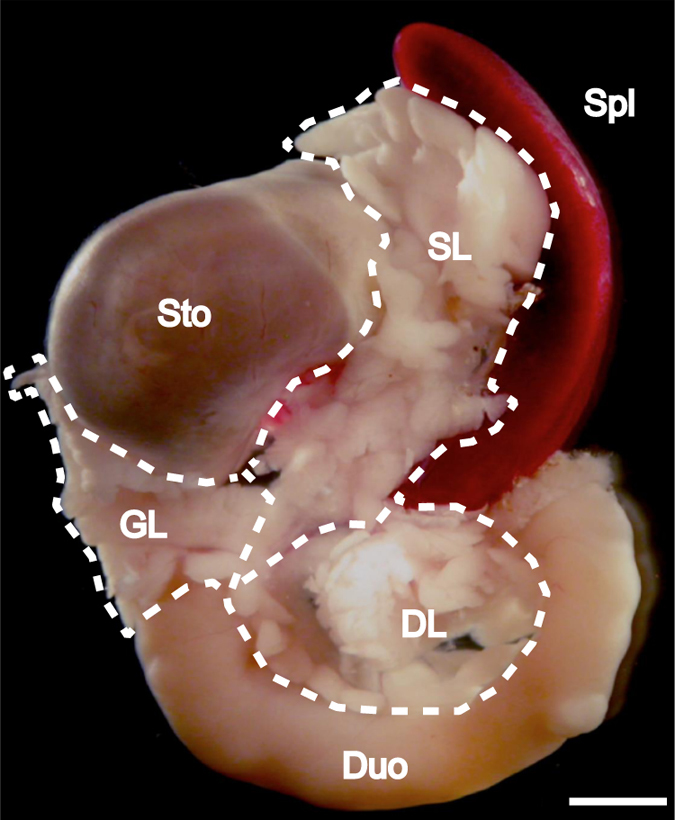
Illustration of lobular separation. The image shows a photomicrograph of a gut segment from a C57Bl/6 mouse at 8 weeks, encompassing the stomach, duodenum, pancreas and spleen. The broken white lines indicate the pancreas and its three primary, gastric, duodenal and splenic lobular compartments. DL, duodenal lobe; Duo, duodenum; GL, gastric lobe; SL, splenic lobe; Spl, spleen; Sto, stomach. Scale bar is 2 mm.

All experiments were conducted in accordance with Umeå University guidelines and national legislation. All animal experiments were approved by the animal review board at the Court of Appeal of Northern Norrland in Umeå, Sweden. Animals were killed by cervical dislocation and pancreata from groups (*n*=5) of *ob/ob*^*Umeå*^ mice and lean control (*ob/+* or *+/+*) littermates were isolated at 4, 8, 17, 26 and 52 weeks of age. The pancreata were fixed in 4% paraformaldehyde (PFA) (Sigma-Aldrich) for 2 h, washed in 1×PBS and the splenic, duodenal and gastric lobes were separated before processing for immunohistochemistry and OPT imaging. The lobular compartments were defined as previously described^13,14^, outlined in Fig. 3).

### Whole mount immunohistochemistry

The staining procedure was performed essentially as described^6^. All washing and dehydration steps were carried out in room temperature (R/T) on a nutator or rotator if not stated otherwise. The pancreata were washed in 50 ml 1×PBS for 2×30 min, dehydrated stepwise (33, 66, 100, and 100%) to 100% Methanol (MeOH), (Scharlau), in 15–30 min steps in a volume of 50 ml. The dehydrated pancreata were stored at −20 ° C until immunohistochemical staining was initiated. In order to increase antibody penetration, the pancreata were freeze-thawed by bringing the samples to −80 °C for 1 h and then back to R/T for 30 min. This procedure was repeated 4 times. The samples were then incubated in a bleaching solution (Modified Dent´s bleach)^4^ consisting of MeOH: Dimethyl sulfoxide (DMSO), (Sigma-Aldrich): Hydrogen peroxide (H_2_O_2_), (Sigma-Aldrich) in a 2:1:3 ratio for 12–24 h at R/T to reduce the endogenous fluorescence. After bleaching the samples were washed 2×30 min in MeOH in volumes of 50 ml and then stepwise rehydrated (33%, 66%, 100%) with TBST (50 mM Tris-HCl pH 7.4, 150 mM NaCl, 0.1% TritonX-100, Merk), 15 min for each step in a volume of 50 ml. The rehydrated pancreata were then blocked in a blocking solution (10% heat inactivated goat serum, 5% DMSO, 0.01% NaN_3_ (Sigma-Aldrich) in TBST) for 12–24 h at R/T. The samples were kept static during this process. The blocking solution was discarded and the samples were incubated in primary antibody staining solution (Guinea Pig anti insulin (DAKO A0564 1:500) diluted in blocking solution for 48 h at R/T. The samples were then washed 3×1 h in 50 ml TBST followed by a O/N wash and another 2×1 h wash prior to secondary antibody incubation. The secondary antibody staining solution (goat Alexa 594 anti-Guinea pig (Molecular Probes A11076 1:500)) was filtered through a 0.45 μm, 25 mm syringe filter (Acrodisk) before incubation for 48 h in R/T. The samples were kept in a dark environment during this process. After antibody incubation, the samples were washed as after primary antibody incubation.

### Sample preparation for OPT scanning and tissue clearing

The pancreata were briefly submerged in MQ (to remove remaining TBST) prior to mounting in filtered (0.8 μm, 150 ml, Nalgene Rapid-Flow sterile disposable filter units with CN membrane, Thermofisher) 1.5% low melting agarose (Seaplaque agarose, Lonza) dissolved in MQ and equilibrated to 38 °C. During mounting, the pancreata were slowly lifted in the agarose by forceps (or inoculation loops) to ensure correct positioning of the specimen at the time point when the agarose solidified. When solidified, the agarose block containing the pancreata were cut out and trimmed, using a scalpel blade, to remove excess agarose. The agarose blocks containing the pancreata were transferred to a 50 ml falcon tube and dehydrated in 100% MeOH at R/T. The blocks were left to equalize to 100% MeOH in a dark environment for 24 h and the MeOH was changed 3 more times. Before transferring the samples to the clearing solution, BABB (Benzyl alcohol (Scharlau), Benzyl Benzoate (Acros organics) in a 1:2 ratio), a ‘BABB test’ was performed on the MeOH from the dehydrated sample to ensure complete dehydration. A small volume of MeOH from the last dehydration step was mixed with fresh BABB solution. The appearance of a white precipitate hereby indicates that additional MeOH changes are necessary. Samples that passed the ‘BABB test’ were transferred into glass containers filled with BABB and equalized O/N in a ventilated and dark environment. The BABB clearing solution were changed every 24 h until phases were no longer visible in the solution and the samples were visually transparent.

### OPT scanning and reconstruction

Centre of mass-axis of rotation (COM-AR)^5^ was performed on all samples prior to scanning to ensure optimal positioning in the scanner. The COM-AR script determines the centre of mass by using two 90° projections, relying on the samples endogenous fluorescence and presents a reference map which is a combination of the specific signal and vertical line indicating the samples centre of mass. The samples were scanned using a Bioptonics 3001 OPT scanner (Bioptonics) modified with an excitation hq565/30x-hc filter (Chroma) and an hq625/50m-hc emission filter (Chroma) detecting the Alexa 594 antibody. 800 projection views (*.tif) were collected throughout the 360° rotation for each channel, The GFP filter was used to depict the anatomy of the organ and the hq625/50m-hc channel the insulin-A-594 staining. Post-OPT acquisition, the projection views underwent two in house developed image processing scripts created in Matlab. These were (1) Discrete Fourier transform alignment (DFTA)^5^, which aligns opposing projections throughout the specimen, resulting in a uniform correction value (A-value) throughout the specimen and (2) Contrast Limited Adaptive Histogram Equalisation (CLAHE)^7^, which increases the sensitivity of OPT imaging for islet detection, helps preserve islet morphology and diminish subjectivity in thresholding for tomographic reconstruction. The tile size for the CLAHE script was 64×64 for all samples. Tomographic reconstruction images (*.bmp) were generated using the NRecon software (V1.6.9.18, Bruker microCT, Belgium) (Tomographic images.zip_Data record A, Data Citation 1).

### 3D volume and iso-surface rendering

Insulin positive β-cell volumes were quantified by 3D iso-surfacing using the Imaris software (v7.7.2, Bitplane, Switzerland). Islet volumes were segmented using the ‘background subtraction (local contrast)’ thresholding option and the intensity threshold was set manually for each pancreas. Iso-surfaced volumes of 10 voxels or less were excluded from the data sets in order to avoid including artefacts from general noise. In this case, 10 voxels translates into a spherical object with a diameter of≤50 μm (The diameter of a β-cell is ≈10 μm) (Isosurfaced Volumes.zip_Data record B, Data Citation 1).

### Data retrieval

Statistical data parameters (Overall, Area, Center of Homogeneous Mass, Ellipsoid Axis A, Ellipsoid Axis B, Ellipsoid Axis C, Ellipsoid Axis Length A, Ellipsoid Axis Length B, Ellipsoid Axis Length C, Ellipticity (oblate), Ellipticity (prolate), Number of Voxels, Position, Sphericity and Volume) were exported from Imaris to Microsoft Excel (Microsoft, USA) spread sheets (*.xls) (Volumetric and spatial statistics.zip_Data record C, Data Citation 1).

### Visual reference images

To create visual reference images, individual images depicting the anatomy and the insulin staining respectively were exported from Imaris and merged using Adobe Photoshop CS6 (Adobe, USA) (Visual reference image.zip_Data record D, Data Citation 1).

### Code availability

The software used for COM-AR^5^, A-value tuning^5^ and CLAHE^7^ used for processing of the OPT projection views were generated in house and is available upon request to the authors subject to a material transfer agreement (MTA). The terms for the MTA follow below:

*In response to the RECIPIENTs request for the material* (1/ implementation scripts for the algorithms presented in Cheddad *et al.*,^5^ IEEE transaction on medical imaging, 2012, https://doi.org/10.1109/TMI.2011.2161590 regarding COM-AR and DFTA, 2/ Software package for implementation of contrast limited adaptive histogram equalization as presented in Hörnblad *et al.*,^7^ Islets, 2011). *The provider asks that the RECIPIENT **agree** to the following terms before the RECIPIENT receives the material.*

1. The MATERIAL is the property of the PROVIDER and is made available as a service to the research community.
2. The MATERIAL will not be further distributed to other without the PROVIDERS written consent. The recipient shall refer any request for the material to the PROVIDER.
3. The recipient agrees to acknowledge the source of the MATERIAL in any disclosure reporting the use of it.
4. THE PROVIDER SHALL NOT BE LIABLE TO THE RECIPIENT FOR ANY LOSS, DAMAGE, COSTS, EXPENSES OR OTHER CLAIMS WHICH ARISE OUT OF OR IN CONNECTION WITH ANY SERVER OR OTHER COMPUTER CRASHING OR FOR THE DELETION, CORRUPTION, LOSS OR REMOVAL OF ANY DATA. IT IS THE RECIPIENTS SOLE RESPONSIBILITY TO TAKE ALL SUCH PRECAUTIONS AS MAY BE NECESSARY OR DESIRABLE TO PROTECT THE RECIPIENTS SYSTEM AND ALL DATA ON IT INCLUDING, WITHOUT LIMITATION, KEEPING COPIES OF ALL RELEVANT MATERIALS, INFORMATION, IMAGES OR DATA AND INSURING AGAINST THEIR LOSS, DAMAGE OR CORRUPTION AND MAKING BACK-UPS.
5. The MATERIAL is provided at no cost.

#### Software versions

Skyscan (V1.3.8, Bruker microCT, Belgium) was used for OPT scanning and the NRecon software (V1.6.9.18, Bruker microCT, Belgium) was used for tomographic reconstruction. The CLAHE^7^ script was set to use a 64×64 tile size. 3D rendering was performed using Imaris (v7.7.2, Bitplane, Switzerland). Objects smaller than 10 voxels were excluded from the data during iso-surfacing (se methods).

## Data Records

**Figure 4:**
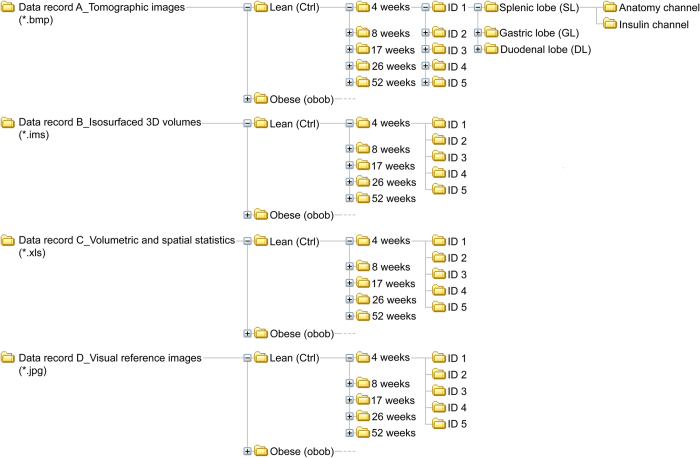
Schematic illustration depicting the layout of the data records (Data records A-D, Data Citation 1). Each Data record folder tree is split at level 1 into ‘lean’ (Ctrl) and ‘Obese’ (*ob/ob*) mice. Level 2 contains the different time points (ages) when the pancreata were isolated. Level 3 represents the individual specimen IDs. Level 4 and 5 only exist for Data record A (Data Citation 1) where level 4 specifies the pancreatic lobe (gastric, duodenal or splenic) and level 5 the visualized channel (anatomy or Insulin respectively).

The data records described in this paper are available at the Dryad Digital Repository (Data records A-D, Data Citation 1) and a schematic illustration of how the records are organised is outlined in Fig. 4. The records describe the full BCM distribution in obese (*ob/ob*) mice and their healthy (*ob/+* or *+/+*) lean controls, including volumetric, spatial and conformational information on individual islet β-cell volumes at 4, 8, 17, 26 and 52 weeks of age.

Data generated by tomographic reconstruction of OPT projection data can be found in ‘Data record A_Tomographic images’ (Tomographic images.zip_Data record A, Data Citation 1). The individual image files are annotated to indicate: phenotype, age (weeks), ID, pancreatic lobe, channel (insulin or anatomy) and sequential stack number, e.g., ‘Ctrl_17w_ID4_SL_Insulin_0123.bmp’.

Data from iso-surface rendering in Imaris can be found in ‘Data record B_Isosurfaced 3D volumes’ (Isosurfaced Volumes.zip_Data record B, Data Citation 1). These files contain both the anatomy and insulin channels and are annotated to indicate phenotype, age (weeks), ID and pancreatic lobe, e.g., ‘Ctrl_17w_ID4_SL.ims’.

Data resulting from data retrieval of 3D volumes can be found in ‘Data record C_Volumetric and spatial statistics’ (Volumetric and spatial statistics.zip_Data record C, Data Citation 1). These files are annotated to indicate phenotype, age (weeks), ID, pancreatic lobe and channel, e.g., ‘Ctrl_17w_ID4_SL_Insulin.xls’ and contain information on:

- Overall sum of volumes, voxels and number of discontinued objects

For all individual objects the following parameters are given (after segmentation):

- Area (the area of the object, calculated as the sum of triangle surfaces.)
- Center of Homogeneous Mass (assuming a homogenous intensity, the centre of homogenous mass is the X, Y, Z coordinates of the object’s centroid (geometric centre)). This value is the same as ‘Position’ (see below). Note, depending on the shape of the object (if the object is concave), the centroid may not be inside the object.
- Ellipsoid Axis A (defines the Vector of the Ellipsoid Axis A).
- Ellipsoid Axis B (defines the Vector of the Ellipsoid Axis B).
- Ellipsoid Axis C (defines the Vector of the Ellipsoid Axis C).
- Ellipsoid Axis Length A (defines the length of the axis A).
- Ellipsoid Axis Length B (defines the length of the axis A).
- Ellipsoid Axis Length C (defines the length of the axis A).
- Ellipticity (oblate) (flattened spheroid, A<B=C)
- Ellipticity (prolate) (elongated spheroid, A=B<C)
- Number of Voxels (number of voxels within the selected object).
- Position (The X, Y and Z coordinates of the surface object. The value is equal to the value of the Center of Homogeneous Mass of the object).
- Sphericity (Sphericity is a measure of how spherical an object is).
- Volume (Volume is a quantification of how much space an object occupies.)

Representative visual reference images for all samples in the study can be found in ‘Data record D_Visual reference images’ (Visual reference image.zip_Data record D, Data Citation 1). These files are annotated to indicate phenotype, age (weeks), ID and lobe, e.g., ‘Ctrl_17w_ID4_SL.jpg.

Metada files indicating the samples provenance, experimental manipulations performed and output can be found as supplementary tables in the corresponding Data record. (README.xlsx_Data record A, Data Citation 1) (README.xlsx_Data record B, Data Citation 1) (README.xlsx_Data record C, Data Citation 1) (README.xlsx_Data record D, Data Citation 1).

## Technical Validation

The optical projection tomography scanner used to acquire the datasets described in this paper was professionally maintained and aligned for optimal imaging conditions prior to dataset acquisition. By the implementation of a custom built specimen mount^6^ and by alignment of the (overlapping) first and last projection image recorded in each scan, we could determine that any potential movements of the specimen during scanning were negligible. Highly experienced animal caretakers with long previous experience of the models performed phenotyping of the utilized animals. In general, the methods and techniques utilized to produce the data has been rigorously tested and have been evaluated in peer reviewed journals^4–7^.

## Usage Notes

The tomographic images (*.bmp) can be imported into most 3D-visualisation software such Imaris (Bitplane, Switzerland), Volocity (PerkinElmer, USA), Drishti (Australian National University, ANUSF VizLab, Canberra, ACT, Australia), etc. When importing the two channels the numerical value of the starting image of the stacks should always be the same for both the anatomy and insulin channels and the stacks must be sorted numerically.

The Imaris files (*ims) contains the full iso-surfaced volumes (anatomy and insulin) and can be used to extract additional information (based on the users Imaris modules) for the total volume or a specific object in the organ as well as for features such as virtual sectioning, movie creation etc.

## Additional Information

**How to cite this article:** Parween, S. *et al.* Spatial and quantitative datasets of the pancreatic β-cell mass distribution in lean and obese mice. *Sci. Data* 4:170031 doi: 10.1038/sdata.2017.31 (2017).

**Publisher’s note:** Springer Nature remains neutral with regard to jurisdictional claims in published maps and institutional affiliations.

## Supplementary Material
